# ‘To prone or not to prone’ in severe ARDS: questions answered, but others remain

**DOI:** 10.1186/cc13893

**Published:** 2014-05-27

**Authors:** Seema S Tekwani, Raghavan Murugan

**Affiliations:** 1 Department of Critical Care Medicine, University of Pittsburgh School of Medicine, 642A Scaife Hall, 3550 Terrace Street, Pittsburgh, PA 15261, USA; 2 The Clinical Research, Investigation, and Systems Modeling of Acute Illness (CRISMA) Center, University of Pittsburgh, 3550 Terrace Street, Pittsburgh, PA 15261, USA

## Expanded abstract

### Citations

Guérin C, Reignier J, Richard JC, Beuret P, Gacouin A, Boulain T, Mercier E, Badet M, Mercat A, Baudin O, Clavel M, Chatellier D, Jaber S, Rosselli S, Mancebo J, Sirodot M, Hilbert G, Bengler C, Richecoeur J, Gainnier M, Bayle F, Bourdin G, Leray V, Girard R, Baboi L, Ayzac L, PROSEVA Study Group: **Prone positioning in severe acute respiratory distress syndrome.***N Engl J Med* 2013, **368:**2159.

### Background

Previous trials involving patients with the acute respiratory distress syndrome (ARDS) have failed to show a beneficial effect of prone positioning during mechanical ventilator support on outcomes. We evaluated the effect of early application of prone positioning on outcomes in patients with severe ARDS.

### Methods

*Objective:* The objective was to evaluate the effect of early application of prone position on mortality in patients with severe ARDS.

*Design:* The PROSEVA group conducted a multicenter, prospective, randomized controlled trial.

*Setting:* Patients with ARDS were recruited from 26 ICUs in France and one ICU in Spain.

*Subjects:* The subjects were critically ill patients admitted to the ICU with respiratory failure requiring mechanical ventilation for severe ARDS. Severe ARDS criteria - an arterial partial pressure of oxygen/fraction of inspired oxygen (PaO_2_/FiO_2_) ratio of less than 150 mm Hg, an FiO_2_ of at least 0.6, a positive end-expiratory pressure of at least 5 cm of water, and a tidal volume of about 6 mL per kilogram of predicted body weight - were confirmed after 12 to 24 hours of mechanical ventilation in the participating ICUs. Subjects were eligible after 12 to 24 hours of stabilization and were randomly assigned to either the prone group or the supine group.

*Intervention:* Four hundred sixty-six patients with severe ARDS underwent prone position ventilation of at least 16 hours or ventilation in the supine position. Patients assigned to the prone group were manually turned in standard ICU beds to the prone position within the first hour of random assignment and were placed prone for at least 16 consecutive hours. Standard ventilator protocols and weaning protocols were implemented for study participants.

*Outcomes:* The primary outcome was the proportion of patients who died from any cause within 28 days after random assignment.

### Results

In total, 237 patients were assigned to the prone group and 229 patients were assigned to the supine group. The 28-day mortality rates were 16% in the prone group and 32.8% in the supine group (*P* <0.001). The hazard ratio for death with prone ventilation was 0.39 (95% CI 0.25 to 0.63). Unadjusted 90-day mortality rates were 23.6% in the prone group and 41% in the supine group (*P* <0.001), with a hazard ratio of 0.44 (95% CI 0.29 to 0.67). The incidence of complications did not differ significantly between the groups, except for the incidence of cardiac arrests, which was higher in the supine group.

### Conclusions

In patients with severe ARDS, early application of prolonged prone-positioning sessions significantly decreased 28-day and 90-day mortality.

## Commentary

Prone position ventilation (PPV) has been shown to improve oxygenation in many animal and human studies of severe acute respiratory distress syndrome (ARDS) [1,2]. The benefits of PPV have been attributed to multiple mechanisms. First, in the supine position, there is more expansion of ventral alveoli compared to dorsal alveoli, and this effect is more profound in patients with ARDS because increased lung weight compresses dorsal alveoli. At the same time, due to the effect of gravity, blood flow is highest in these dorsal lung fields causing a ventilation perfusion mismatch. When the patient is placed in the prone position, the pressure gradient from ventral to dorsal regions of the lung is reduced and trans-pulmonary pressures are more uniformly distributed, making ventilation more uniform [3]. Second, with PPV, improved ventilation in previously dependent areas in the dorsal region now matches the sustained higher dorsal perfusion, thus significantly reducing the ventilation/perfusion mismatch [4]. Third, PPV reduces alveolar collapse and hyperinflation by decreasing lung compression by caudal displacement of abdominal organs and also decreasing the weight of the heart on the left lower lobe of the lung parenchyma [5]. Fourth, PPV improves bronchial drainage and aids secretion clearance by the effects of straightening the larger airways with gravity assistance [6]. Finally, PPV decreases chest wall compliance, thus improving functional residual capacity [7].

Many clinical trials have been conducted to assess the clinical benefits of PPV in patients with ARDS. Gattinoni and colleagues [8] conducted the first clinical trial and demonstrated improvement in oxygenation in patients on PPV. Similar results of improved oxygenation have been found in another study [9]. However, none of these earlier studies has been able to show any difference in mortality. Proponents of PPV feel that the initial studies were fraught with poor study design as they did not clearly define study protocols and enrolled a heterogeneous group of patients with less severe ARDS and patients with non-ARDS-related hypoxemic respiratory failure [10]. Other criticisms include late implementation of PPV after onset of ARDS and shorter duration of PPV (about 6 to 8 hours per day).

Subsequent randomized controlled trials attempted to enroll patients earlier (Guérin and colleagues [9]) and for longer duration [11]. However, these studies were underpowered to detect statistically significant effects on mortality [9]. Taccone and colleagues [12] enrolled patients with arterial partial pressure of oxygen/fraction of inspired oxygen (PaO_2_/FiO_2_) ratios of less than 100, with the intervention group receiving PPV for 20 hours per day. However, this trial did not show any mortality benefit; on the contrary, it showed a significant increase in adverse events with PPV [12]. Nevertheless, meta-analysis and subgroup analyses of patients with severe ARDS have shown a trend toward mortality benefit [13]. Thus, PPV remained a grade 2B recommendation in the 2012 Surviving Sepsis Guidelines and in the primary role as a rescue therapy in patients with severe ARDS [14].

In this multicenter clinical trial, Guérin and colleagues (see ‘Citation’ above) examined whether PPV has any beneficial effects in the sickest of ARDS patients with severe hypoxemia when PPV was initiated within 24 hours after onset of ARDS and sustained for longer duration of at least 16 hours per day. Strengths of the study include a well-conducted multicenter trial, inclusion of a homogenous group of patients with severe ARDS, use of lung protective ventilation in both arms, and use of standard ventilator weaning protocols. The authors were able to show a remarkable benefit of PPV with a huge effect size of 51% relative risk reduction (RRR) in mortality at 28 days with a number needed to treat of 6. This mortality reduction persisted at 90 days (23.6% versus 41%; *P* <0.001).

Important limitations of the study include imbalances in baseline characteristics with more severely ill patients enrolled in the control group, as evidenced by higher Sequential Organ Failure Assessment scores and increased vasopressor requirement, compared with the PPV group. This difference between the groups could have biased the result toward benefit for the PPV group. In addition, the PPV group was noted to have received more neuromuscular blockers, which may have magnified the treatment effect as use of neuromuscular blockers has been shown to improve mortality in patients with ARDS [15]. This makes us further question the care received for the supine patients as blinding was impossible and may have influenced the care received by the control group. Recent meta-analysis on PPV predicted a mortality benefit of approximately 16% RRR in severe ARDS patients with a PaO_2_/FiO_2_ ratio of less than 100, but the large effect size of more than 50% RRR in the PROSEVA (Proning Severe ARDS Patients) trial has not been reported with any critical care intervention questioning the biologic plausibility of such a huge benefit [13]. This finding might have resulted as a consequence of possible inflated control group mortality (32%) as current ARDS studies report much lower mortality rates.

It is important to note that the study was conducted in an environment of practitioners who are highly skilled and trained in prone ventilation. Management of the prone patient is an acquired skill and is technically challenging as detailed in the video accompanying the article. Widespread implementation of PPV requires the health-care system to be equipped with additional resources like training and education, PPV-specific protocols, and expertise to avoid serious adverse events in order to replicate the results and low complication rate seen in the PROSEVA study. The PROSEVA trial also suggests initiating PPV for all moderate to severe ARDS (FiO_2_ of at least 0.6 and positive end-expiratory pressure of at least 5), which would require initiating PPV in many patients with ARDS and hence tremendous education efforts and resources for widespread implementation.

## Recommendation

Although the results of the PROSEVA trial need to be replicated, the study findings suggest that using PPV earlier, more often, and for longer durations in patients with severe ARDS results in better outcomes than does offering PPV as a rescue maneuver and a last-ditch effort.

## Abbreviations

ARDS: Acute respiratory distress syndrome; FiO2: Fraction of inspired oxygen; PaO2: Arterial partial pressure of oxygen; PPV: Prone position ventilation; PROSEVA: Proning severe ARDS patients; RRR: Relative risk reduction.

## Competing interests

The authors declare that they have no competing interests.
